# Multimodal teaching methods for students in dentistry: a replacement for traditional teaching or a valuable addition? A three-year prospective cohort study

**DOI:** 10.1186/s12909-023-04377-z

**Published:** 2023-06-02

**Authors:** Gabriella Gatt, Nikolai J. Attard

**Affiliations:** 1 Department of Child Dental Health and Orthodontics, Faculty of Dental Surgery, University of Malta, Msida, Malta; 2 Department of Oral Rehabilitation and Community Care, Faculty of Dental Surgery, University of Malta, Msida, Malta

**Keywords:** Dental Research, Students, Dental, Education, Academic performance, Teaching, Educational Technology

## Abstract

**Background:**

This student-centred prospective cohort study evaluated the impact of multimodal teaching methods on student performance in the theoretical domain of dental studies.

**Methods:**

Dental students answered anonymous questionnaires indicating their preferences and opinions three times over three consecutive academic years. Data collected included gender, course, year of study and most frequent and preferred learning modality. Survey responses from Google Forms were analysed with SPSS 20.0 software (IBM Company, Chicago, IL, USA). Scale responses were tested with the Mann-Whitney U test against gender, program and year of study. Grades obtained from structured examinations held in the third academic year were analysed using the Wilcoxon Signed Rank Test according to the teaching method employed. The level of statistical significance was set at p < 0.05.

**Results:**

The response rate was high (> 80%) throughout the study. Acceptance of online modalities increased over time (Kruskal-Wallis test, p < 0.001) and 75% of students requested that online teaching modalities be maintained. Significant differences in gender, program of study, year of study and discipline taught were observed (Mann-Whitney test, p < 0.05). Females differed from males by favouring online modalities and face-to-face lectures, respectively, and clinical year students opted to retain pre-recorded online lectures. Recorded lectures resulted better for teaching core knowledge (Wilcoxon Signed Rank Test, p = 0.034), while face-to-face lectures were better for teaching applied knowledge (Wilcoxon Signed Rank Test, p = 0.043). Student responses to open-ended questions identified the need for a blended approach with in-person lecturing as an opportunity to socialise and avoid mental health issues. Although preferences varied, students showed a willingness to influence their learning and changes in curriculum, a predilection for self-directed learning and the need for freedom in engaging with resources and content.

**Conclusions:**

In the context of this study, online teaching modalities resulted in comparable examination performance and improved student satisfaction. This highlights the need for a blended approach to teaching.

## Background

Adult learning theory postulates that adult learners’ needs and motivations differ from those of younger learners. This theory suggests that traditional classroom teaching is not ideal for self-motivated students over the age of 18, who tend to be self-directed learners [1] actively participating in the planning and input of learning. It is suggested that adult learners be provided with opportunities to influence curriculum [2] and changes in the andragogy of dentistry [3, 4].

Previous studies have shown that computer-aided, self-instructional programs for teaching are more or equally as effective as other methods of instruction [5]. The provision of material through a virtual learning environment (VLE) platform using media that includes videos, presentations, or text to be accessed at will, before the class discussion, has been shown to provide all types of learners an equal opportunity to succeed [6] whilst allowing them the autonomy of self-paced learning. Although learning preferences have been shown to be influenced by gender [7, 8], pre-clinical or clinical level of study [9], and the type of program of study [10, 11], further investigation is still required in the area of dentistry as these studies[5–10] do not provide a complete overview due to the inclusion of non-dentistry students, the inclusion of graduated professionals, low response rates and may have not explored the diverse realities of preclinical and clinical students.

Allowing dentistry students to participate in the process of change of faculty teaching modalities was seen as an opportunity to allow for improved motivation and learning and, ultimately, better application of knowledge. Guided by the principles of Adult Learning Theory, the SQUIRE-EDU process of implementing and reporting educational improvement [12], and ongoing student feedback on their educational experiences, preferences and challenges, the faculty embarked on a project to assess, modify and reassess its teaching methods to evaluate the impact of various multimodal teaching methods on student performance in the theoretical domain.

## Methods

This study evaluated the preferences for various learning modalities and the resulting academic performance of dentistry students in the theoretical domain of dental studies over three academic years.

The prospective cohort study with an exploratory sequential design included the entire undergraduate dental student population. These included master in dental surgery (MDS) (two pre-clinical years, three clinical years), dental hygiene (DH) bachelor degree program (one pre-clinical year, two clinical years) and dental technology (DT) bachelor degree program (laboratory based) students. The MDS degree, although a 5-year long course, is still considered as an undergraduate degree that leads to a professional warrant in dentistry.

Clinical and behavioural skills are taught via pre-clinical simulation lab teaching, clinical patient treatment sessions and outreach activities. Didactic teaching is carried out by combining in-person (face-to-face/F2F) lectures and tutorials, synchronous online lectures and tutorials, uploaded online material and asynchronous online pre-recorded lectures (REC). For REC lectures, lecturers could use either presentation software applications or video platforms uploaded on the university VLE.

**Fig. 1:**
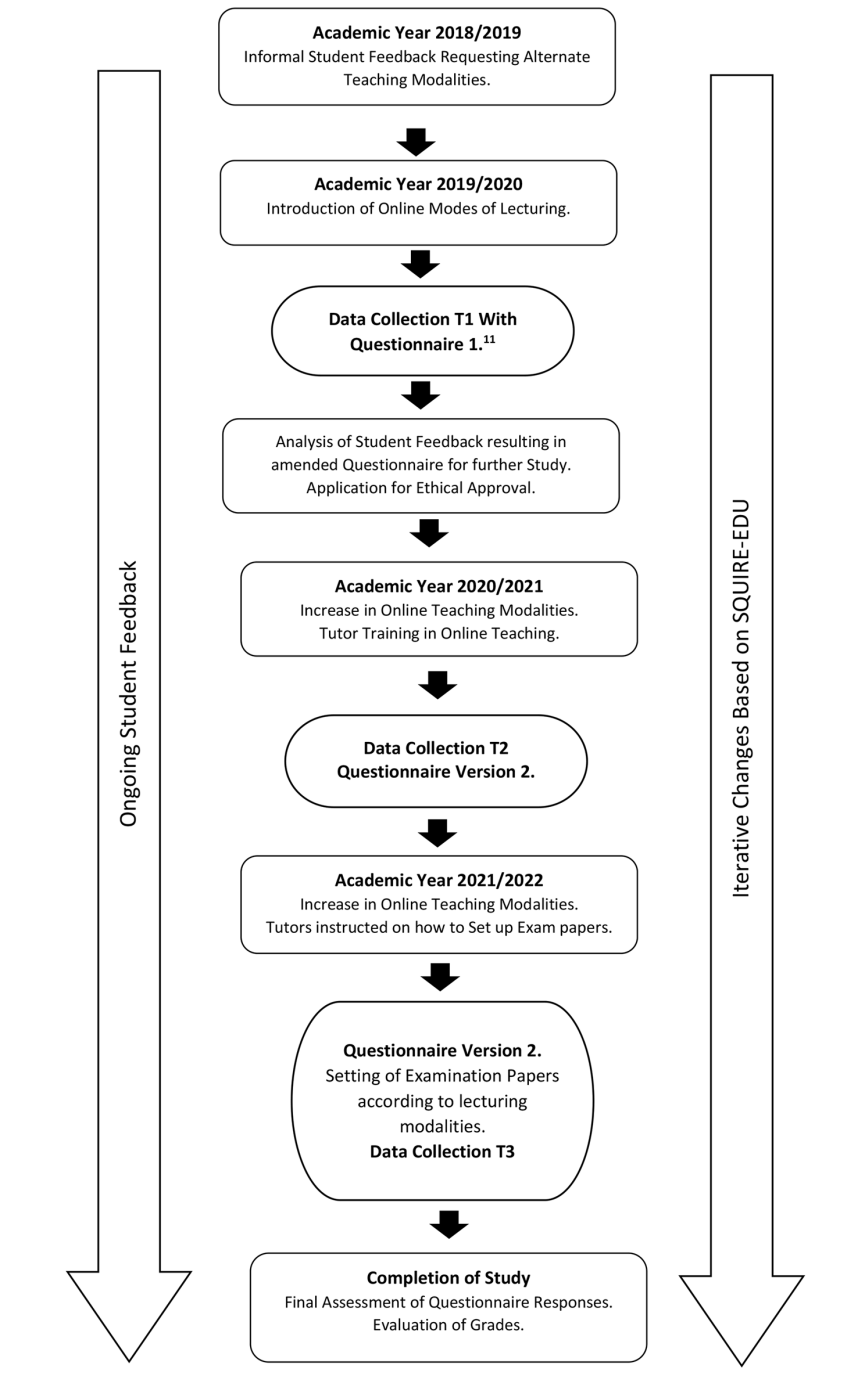
Flow Diagram of Study Method

The Flow diagram in Fig. 1. displays the design of the study. In the academic year 2019/2020, in response to informal student feedback received relating to the previous year (2018/2019), academic lecturing staff were encouraged to move away from providing exclusively in-person face-to-face (F2F) classroom teaching and to explore online modes of teaching. The university offers technology support by providing webinars, seminars, and online material to support lecturers in using new teaching modalities. Tutors were provided with step-by-step instructions for organising online teaching via presentations and offered in-person/online personalised support. Based upon questionnaire (Q1) responses [11], the faculty proceeded with furthering online methods of teaching and studying the outcomes of this intervention. Both research protocols describing the analysis of student perspectives over time of the various modalities of teaching, both online and in-person, being offered were approved by the Faculty Research Ethics Committee and subsequently by the University Research Ethics Committee. A draft of a questionnaire, based on that of a previous survey [13], was adapted to the local context and then discussed with two senior academics and piloted with six students selected equally from the pre-clinical and clinical class cohorts of both the MDS course and the BSc course.

Suggestions provided were discussed and considered. The questionnaire (Q2) was circulated amongst the students at the end of each of the following two consecutive academic years (2020/2021, 2021/2022) over Google Forms documents. The questionnaires allowed participants to skip any questions and to withdraw at any stage. Participants were informed that data collected would be used for research purposes and consent was obtained by completing the first question of the online form.

The questionnaires first asked for students to indicate their gender, year of training and program of study. They were asked to identify the form of teaching they were exposed to most over the previous year. They were also requested to rate their overall experience of online learning as either ‘very good’, ‘good overall’, ‘same – makes no difference’, ‘bad’ or ‘very bad’. The questionnaire (Q2) also included two open-ended questions that allowed students to express their thoughts about online learning and improvements for the faculty to consider.

During the second (T2) and third (T3) academic years (2020/2021; 2021/2022 respectively), a blend of lecture modalities across all the departments were again delivered with an increased focus on online lectures. Tutors were instructed by the Dean and Heads of Department to plan lectures by distributing them over the various teaching modalities. The various lecture dissemination modes were recorded by the faculty office. Examination type, knowledge being assessed (core or applied), class, gender and subject/discipline examined were mapped out.

At the end of the third academic year (T3 -2021/2022) the student grades obtained for the academic year were analysed according to modality of teaching (in-person\F2F lectures and tutorials versus Rec lectures), discipline (prosthodontics, operative dentistry, endodontics, periodontics, preventive dentistry, special care dentistry, orthodontics), core versus applied knowledge being assessed, assessment type (Short answer questions [SAQ] versus multiple choice questions [MCQ]), gender and level of training (Pre-clinical versus clinical).

Tutors were requested to (1) identify how the topics set in examination papers were delivered (Rec or F2F) and examined (SAQ or MCQ) format, and (2) set examination papers that included core theoretical knowledge (CK) presenting principles and current facts, and applied knowledge (AK) presented in case-based learning formats of the subject.

The responses collected were displayed on Google Sheets and tabulated into Microsoft Excel© (Microsoft Corporation 2018).

### Statistical analysis

Statistical tests were carried out with the aid of SPSS 20.0 software (IBM Company, Chicago, IL, USA).

Analyses of continuous dependent variables were conducted with the Mann-Whitney U test, the Related Samples Wilcoxon Signed Rank Test, and the Kruskal Wallis test. The Chi-square test was used for categorical dependent variables. Independent variables included lecture delivery type, gender, type of dental course, year of training and subject discipline. The level of statistical significance was set at p < 0.05.

## Results

The response rate in each of the three years was 90% (n = 88), 83% (n = 98) and 97% (n = 78) respectively of the total student body.

### Cohort demographics

The female to male ratio was 69:31. The mean age at the end of the study was 21 (SD ± 1.85) years. There were no significant differences in gender distribution for degree course (χ^2^(2, n = 97) = 4.952, p = 0.084), year of study (χ^2^(2, n = 250) = 1.67, p = 0.195), and lecture delivered (χ^2^(18, n = 97) = 20.67, p = 0.296).

### Lecture type

**Fig. 2:**
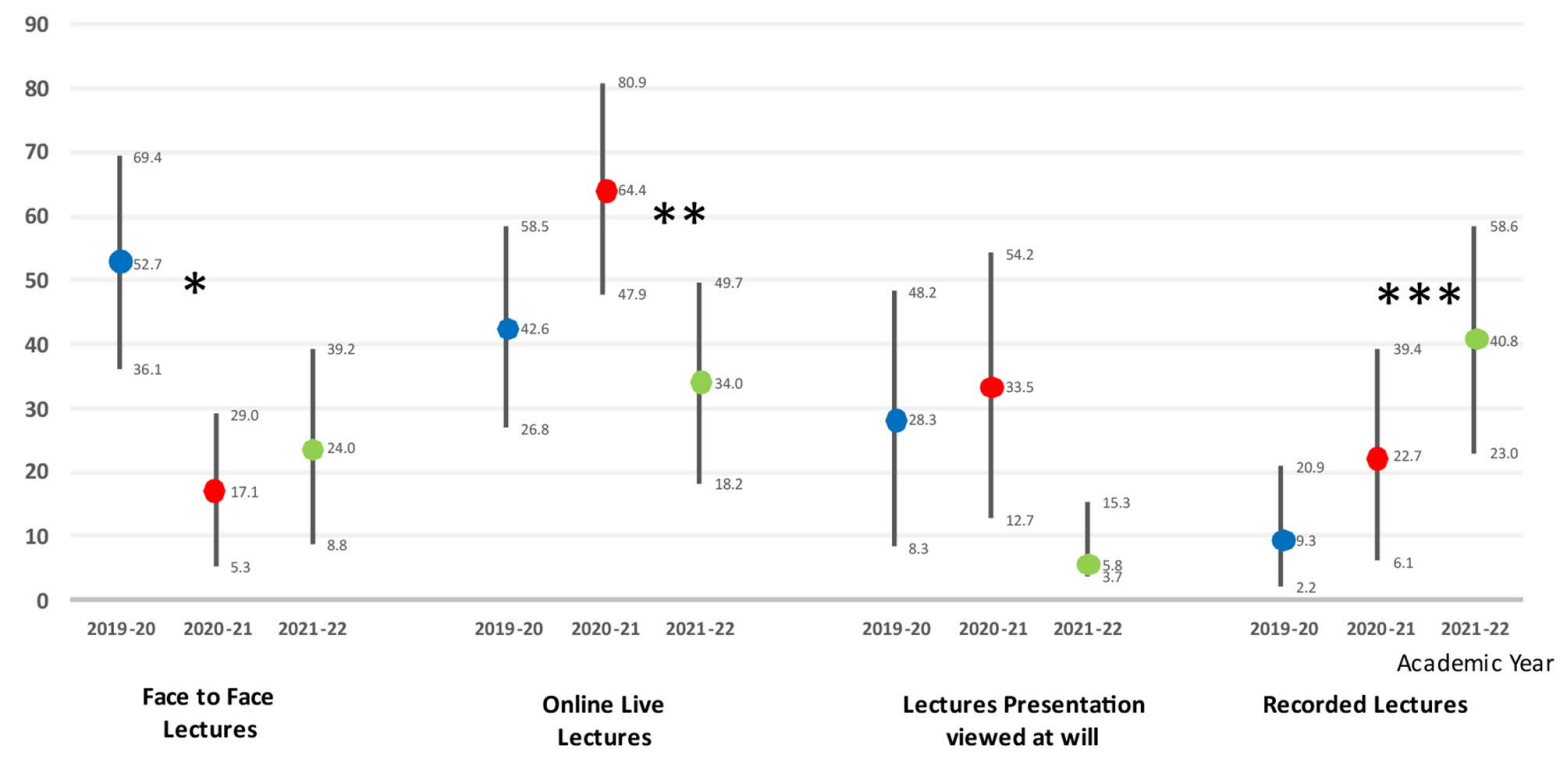
Mean Rate &95% CI of Delivery of Lectures over the study period Kruskal Wallis Test: Online Live Lectures p = 0.018 ** Mann Whitney U-test p = 0.005 (2020-21 > 2021-22) Kruskal Wallis Test: Presentations viewed at will: p = 0.06 Kruskal Wallis Test: Recorded lectures p = 0.004 *** Mann Whitney U-test p = 0.001 (2021-22 > 2019-20) Kruskal Wallis Test: Face to Face Lectures p = 0.007 * Mann Whitney U-test p = 0.003 (2019-20 > 2020-21)

The trends of change in lecture modalities over the three academic years are displayed in Fig. 2. Most lecturers (54%) chose Microsoft PowerPoint/Keynote as the presentation software for their pre-recorded lectures whereas 25% opted for Panopto. Lectures were disseminated by uploading on the Virtual Learning Environment (VLE) platform (46%), YouTube (42%) via email (17%) or via other routes (33%), with some tutors opting for more than one method of distribution. Pre-recorded online lectures with online or in-person tutorials and then a blend of presentations and/or other printed materials via VLE platform were also provided. Tutorials were provided either sequentially slotted between pre-recorded lectures (30%), only when students requested them (30%) or not provided at all (18%). 22% of lecturers provided feedback either in person or via email. 64% of tutorials were held via an online platform, the rest were held in-person in a classroom setting.

### Student feedback

**Fig. 3:**
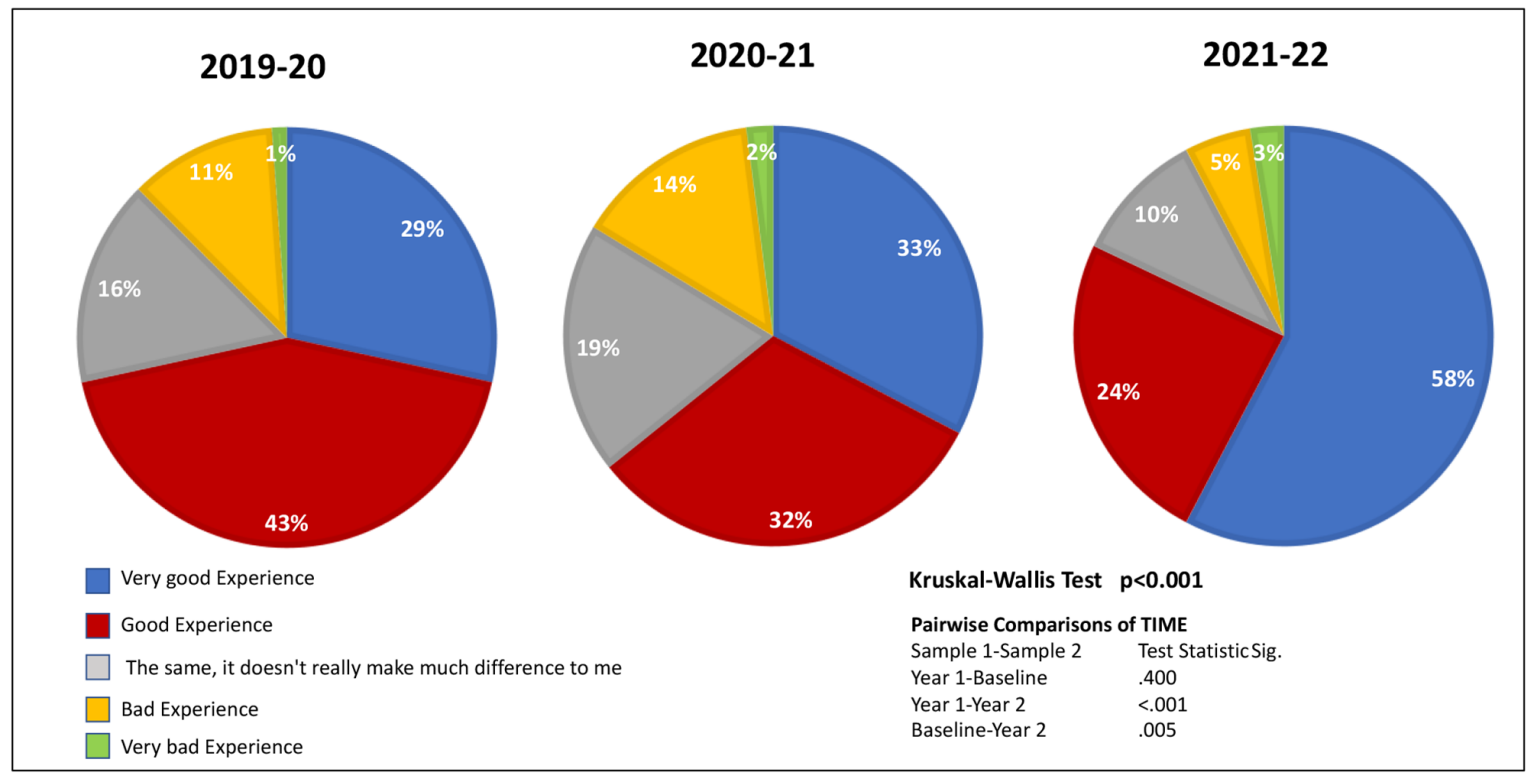
Student response for rate with the overall experience with online lecturing (% agreement)

**Fig. 4:**
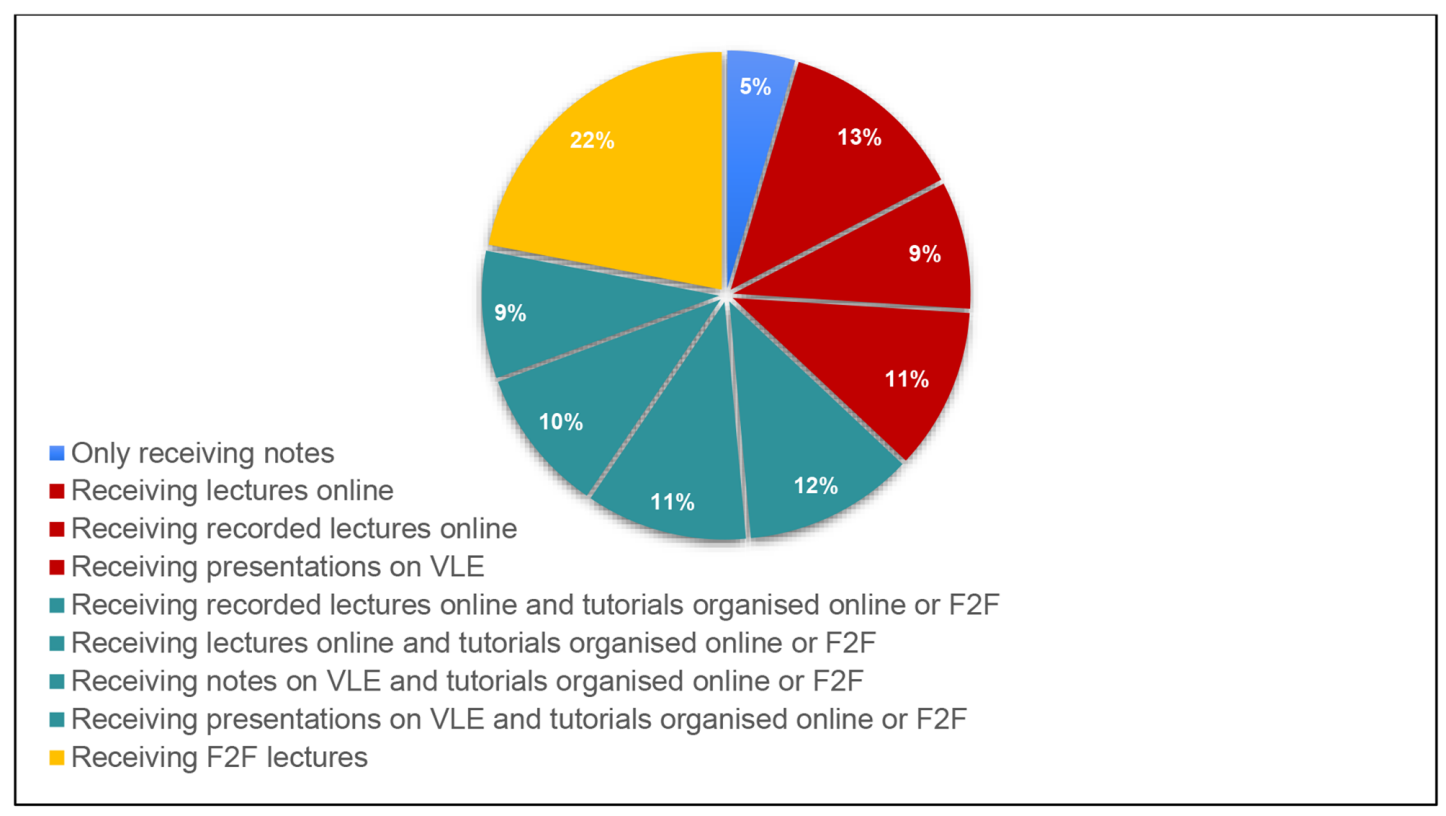
Student preferences for Teaching Methods

At the first questionnaire (Q1) (2019/2020), 74.5% of the student body agreed that online modes of teaching should be retained Fig. 3 presents the change in student responses regarding their experiences with online lecturing over the three academic years. Figure 4 depicts their preferences for teaching modalities at the end of the third year. 75% of all students preferred a variation including an online modality; 33% preferring online only and 42% opting for one of the variations of a blended approach. As for the rest, 22% preferred F2F and 5% notes only.

Further analysis of these findings revealed significant differences for gender, course of study and year of study as shown in Table 1.

**Table 1:** Group comparisons for preference of learning modalities

Online Learning Options	Gender		Course		Clinical/ Preclinical Year		Change Over Time
	**T2**	**T3**	**T2**	**T3**	**T2**	**T3**	
Receiving F2F lectures	**0.003** (M > F)	p > 0.05	p > 0.05	p > 0.05	**0.001** (NC > C)	p > 0.05	**0.05** (T2 > T3)
Receiving lectures online	p > 0.05	p > 0.05	p > 0.05	p > 0.05	p > 0.05	p > 0.05	p > 0.05
Receiving lectures online and tutorials organised online or F2F	p > 0.05	p > 0.05	p > 0.05	p > 0.05	p > 0.05	p > 0.05	p > 0.05
Receiving Rec lectures online	**0.025** (F > M)	p > 0.05	p > 0.05	p > 0.05	**0.021** (C > NC)	p > 0.05	p > 0.05
Receiving Rec online and tutorials organised online or F2F	p > 0.05	p > 0.05	p > 0.05	p > 0.05	p > 0.05	p > 0.05	p > 0.05
Receiving presentations on VLE	**0.038** (F > M)	p > 0.05	**0.011** (B > MDS)	p > 0.05	p > 0.05	p > 0.05	p > 0.05
Receiving presentations on VLE and tutorials organised online or F2F	p > 0.05	p > 0.05	p > 0.05	p > 0.05	p > 0.05	p > 0.05	p > 0.05
Receiving notes on VLE and tutorials organised online or F2F	p > 0.05	p > 0.05	p > 0.05	p > 0.05	p > 0.05	p > 0.05	p > 0.05
Only receiving notes	p > 0.05	p > 0.05	**0.013** (B > MDS)	p > 0.05	p > 0.05	p > 0.05	p > 0.05

Brackets denote group preference

M = Males, F = Females; NC = preclinical years, C = clinical years; B = Bachelor degree, MDS = Dental degree

Statistical Test: Mann-Whitney U Test

Group comparisons of students’ agreement with the statements on diverse aspects of online learning modalities are presented in Table 2. Significant differences were observed, with clear distinctions identified between clinical and preclinical years of study. Table I and Table 2 also display how student preferences changed over time (T2 versus T3).

**Table 2:** Student agreement with statements about online learning

	Gender		Course			Clinical/ Preclinical			Change between Time	
	**T2**	**T3**	**T2**	**T3**	**T2**		**T3**			
Lecturing sessions are more suitable delivered with distance learning modalities	> 0.05	> 0.05	> 0.05	> 0.05	**0.005** (C > NC)		**0.008** (C > NC)	> 0.05		
Clarification sessions are more suitable delivered with distance learning modalities	> 0.05	> 0.05	> 0.05	> 0.05	> 0.05		> 0.05	> 0.05		
Clarification sessions are more suitable delivered in F2F meetings	**0.040** (M > F)	> 0.05	> 0.05	> 0.05	**0.007** (NC > C)		**0.022** (NC > C)	> 0.05		
The flipped classroom model, in which course material is first provided online prior to the instructors addressing the material during class-time, should be implemented in the faculty	> 0.05	> 0.05	> 0.05	> 0.05	> 0.05		> 0.05	> 0.05		
I do not experience any IT connection problems during online learning	> 0.05	> 0.05	**0.030** (B > MDS)	> 0.05	> 0.05		> 0.05	**0.005** T3 > T2		
I do not experience anxiety if I am asked questions during online learning	> 0.05	> 0.05	> 0.05	> 0.05	**0.010** (C > NC)		> 0.05	> 0.05		
I have more time to go through and read learning materials before group discussion with online learning	> 0.05	> 0.05	> 0.05	> 0.05	**0.045** (C > NC)		> 0.05	> 0.05		
I have more time to revise all of the learning materials after class with online learning	> 0.05	> 0.05	> 0.05	> 0.05	**0.021** (C > NC)		> 0.05	> 0.05		
I like online learning more than classroom learning	**0.019** (F > M)	> 0.05	> 0.05	> 0.05	**< 0.000** (C > NC)		**0.030** (C > NC)	**0.008** T3 > T2		
I study more efficiently with online learning resources	> 0.05	> 0.05	> 0.05	> 0.05	**< 0.000** (C > NC)		> 0.05	**0.032** T3 > T2		
Online learning motivates me to prepare learning materials for group discussion/s and to self-directed learning	> 0.05	> 0.05	> 0.05	> 0.05	**0.002** (C > NC)		> 0.05	**0.031** T3 > T2		
Online lecturing should be implemented and maintained in the next academic year	> 0.05	> 0.05	> 0.05	> 0.05	**< 0.000** (C > NC)		> 0.05	**0.003** T3 > T2		
Online learning gives similar learning satisfaction to classroom learning	> 0.05	> 0.05	> 0.05	> 0.05	**< 0.000** (C > NC)		**0.032** (C > NC)	**0.031** T3 > T2		
Communication with lecturers and fellow students is easier with online platforms	> 0.05	> 0.05	> 0.05	> 0.05	**0.035** (C > NC)		> 0.05	> 0.05		
Recorded lectures allows the student to listen to the lecture at a convenient time when the student is fully focused	> 0.05	> 0.05	**0.001** (MDS > B)	> 0.05	**0.026** (C > NC)		> 0.05	> 0.05		
Recorded lectures resources allows the faculty to better utilise the contact time with students for clinical/preclinical skills sessions	> 0.05	> 0.05	**< 0.0001** (MDS > B)	> 0.05	> 0.05		**0.035** (C > NC)	> 0.05		
The provision of lectures in between, or after, clinical/preclinical skills labs sessions is too tiring	> 0.05	> 0.05	> 0.05	> 0.05	**< 0.0001** (C > NC)		> 0.05	> 0.05		

Brackets denote group preference

M = Males, F = Females; NC = preclinical years, C = clinical years; B = Bachelor degree, MDS = Dental degree

Statistical Test: Mann-Whitney U Test

### Student grades

Table 3 displays the *in between group* results for type of lecture delivery (REC or F2F lecture), assessment type (SAC or MCQ), type of knowledge tested (CK or AK) obtained when comparing gender and level of training.

**Table 3:** Group differences in Correct Short and Multiple-Choice Questions between Genders and Years of Clinical Training

Lecture Delivery & Question	GENDER	MEAN RANK	*p-value*	CLINICAL TRAINING	MEAN RANK	*p-value*
Rec SAQ	Females	**124.81**	**0.049**	Preclinical	125.02	0.165
	Males	106.83		Clinical	112.62	
F2F SAQ	Females	113.5	0.567	Preclinical	133.05	**< 0.001**
	Males	108.46		Clinical	93.84	
CK-REC SAQ	Females	**92.27**	**0.032**	Preclinical	72.08	**0.003**
	Males	75.52		Clinical	**95.19**	
CK-F2F SAQ	Females	82.96	0.316	Preclinical	83.28	0.448
	Males	75.49		Clinical	77.67	
AK-REC SAQ	Females	89.97	0.175	Preclinical	86.62	0.894
	Males	79.36		Clinical	85.59	
AK-F2F SAQ	Females	80.51	0.863	Preclinical	78.33	0.7
	Males	79.22		Clinical	81.18	
Rec MCQ	Females	8.79	0.684	Preclinical		N/A
	Males	7.63		Clinical		
F2F MCQ	Females	8.96	0.521	Preclinical		N/A
	Males	7.13		Clinical		
CK-REC MCQ	Females	8.75	0.77	Preclinical		N/A
	Males	7.75		Clinical		
CK-F2F MCQ	Females	8.83	0.684	Preclinical		N/A
	Males	7.50		Clinical		
AK-REC MCQ	Females	9.08	0.446	Preclinical		N/A
	Males	6.75		Clinical		
AK-F2F MCQ	Females	8.67	0.862	Preclinical		N/A
	Males	8.00		Clinical		

SAQ- Short Answer Question; MCQ- Multiple Choice Answer Question; CK- Core Knowledge; AK- Applied Knowledge; Rec- Delivery of Lecture through Recorded Modalities

F2F- Face-to-face delivery of Lecture; IQR- Inter-Quartile Range (denoted in Brackets) Statistical Test: Mann Whitney U Test

Table 4 displays the same comparisons for *within group* results for gender (male and female) and level of training (preclinical and clinical).

**Table 4:** Within group differences in Correct Answers for Short and Multiple-Choice Questions according to Gender and Year of Clinical Training

Lecture Delivery & Question Type	GLOBAL				GENDER							CLINICAL TRAINING								
		**Median & IQR**	***p-value***	**Females Median & IQR**		***p-value***		**Males Median & IQR**		***p-value***			**Preclinical Median & IQR**		***p-value***		**Clinical Median & IQR**		***p-value***	
Rec SAQ		76.00 (66.00–84.00)	0.110	77.78 (68.00-86.17)		0.794	74.00 (61.00–80.00)		**0.034**		76.00 (68.00-86.67)			**0.001**		75.00 (64.00–82.00)		0.368		
F2F SAQ		80.00 (64.00–88.00)		80.00 (64.00-88.08)			**78.17** **(64.25–87.67)**				**84.00** **(73.33–86.59)**					75.00 (58.00–84.00)				
CK-REC SAQ		75.00 (62.50-90.91)	**0.034**	**80.00** **(65.00-93.33)**		**0.019**	73.40 (60-38-86.20)		0.621		73.33 (55.21–86.59)			0.439		**80.00** **(66.67–93.33)**		**0.001**		
CK-F2F SAQ		**80.00** **(60.00–90.00)**		76.47 (60.00–90.00)			79.20 (47.20-88.46)				84.61 (37.50-93.33)					79.00 (60.00-87.20)				
AK-REC SAQ		75 (56.67–88.89)	**0.043**	76.47 (60.00–90.00)		0.330	72.20 (50.00- 88.22)		0.05		75.96 (35.85-92.00)			0.061		75.00 (61.54–85.71)		0.264		
AK-F2F SAQ		**80** **(60.00-91.67)**		75.00 (60.00-92.72)			**80.0** **(53.33- 90.00)**				80.00 (48.75–91.67)					75.00 (60.36–90.20)				
Rec MCQ		60.52 (52.63–68.42)	**0.03**	60.52 (52.63–72.36)		0.071	60.52 (46.05–67.10)		0.273		60.53 (52.63–68.42)			**0.030**				N/A		
F2F MCQ		**72.72** **(65.91–81.82)**		72.72 (65.91–81.82)			72.73 (59.09–79.54)				**72.72** **(65.90-81.82)**									
CK-REC MCQ		69.23 (55.77–84.61)	0.148	73.07 (55.77–84.62)		0.239	69.23 (57.69–80.77)		0.465		69.23 (55.78–84.61)			0.148				N/A		
CK-F2F MCQ		80 (70.00–90.00)		80 (70.00–90.00)			80.00 (57.50–87.50)				80.00 (70.00–90.00)									
AK-REC MCQ		40.62 (22.40-58.84)	0.658	41.67 (33.33-50.00)		0.723	33.33 (20.83–45.83)		0.713		33.33 (33.33-50.00)			0.659				N/A		
AK-F2F MCQ		31.25 (13.03–49.47)		33.3 (17.36–49.24)			20.00 (0.0–75.00)				0.00 (0.00-100.00)									

SAQ- Short Answer Question; MCQ- Multiple Choice Answer Question; CK- Core Knowledge; AK- Applied Knowledge; Rec- Delivery of Lecture through Recorded Modalities

F2F- Face to face delivery of Lecture; IQR- Inter-Quartile Range (denoted in Brackets)

Statistical Test: Related-Samples Wilcoxon Signed Rank Test

Question Type - On comparing males to females, the only significant finding was that females (Md = 124.81) scored better than males (Md = 106.83) when answering SAQs in general (U = 5502.5, z = -1.969, p = 0.049), and that female students answering SAQ for core knowledge (Md = 92.27) following recorded lectures scored better than male students (Md = 75.52) (U = 2753.5, z = -2.144, p = 0.032). No other differences were noted between genders for question type, content or lecture delivery type. Overall, students answering SAQ showed no statistically different results whether following recorded or F2F lectures. On the other hand, better global MCQ results were obtained following F2F lectures (Wilcoxon Signed Rank Test z = 2.172, n = 116, p = 0.030). Similar results were obtained when answering core knowledge, (Wilcoxon Signed Rank Tests z = -2.125, n = 144, p = 0.034) and similarly applied knowledge, (Wilcoxon Signed Rank Tests z = 2.022, n = 144, p = 0.043) topics with SAQ. There was no difference in grades for CK or AK following either recorded or face-to-face lectures when answering via MCQs.

### Gender

In general, females were not affected by the mode of lecture delivery however scored better when answering SAQs following recorded lectures (Related-Samples Wilcoxon Signed Rank Test, (MD = 80) z = -2.348, n = 83, p = 0.019) as opposed to face-to-face lectures (MD = 76). Lecture delivery did not affect MCQ type assessments. Males on the other hand generally fared better with face-to-face lectures (Related-Samples Wilcoxon Signed Rank Test, (MD = 78), z = 2.116, n = 86, p = 0.034) as compared to recorded lectures (MD = 74) and when answering SAQ type assessments for applied knowledge (Related-Samples Wilcoxon Signed Rank Test, (MD = 80), z = 1.955, n = 61, p = 0.05) as compared to recorded lectures (MD = 72). Again, type of lecture did not affect MCQ type assessments.

### Level of training

Students in the preclinical years scored better for both SAQ (Related-Samples Wilcoxon Signed Rank Test, (MD = 84), z = 3.348, n = 100, p < 0.001) and MCQ (Related-Samples Wilcoxon Signed Rank Test, (MD = 72), z = 2.172, n = 61, p = 0.030) following F2F lectures as compared to recorded lectures (Md = 76, MD = 60.5, respectively) with no difference observed as to whether it was CK or AK being assessed. Clinical year student scores showed no overall differences however, scores for CK following recorded lectures (Md = 80) were significantly higher than following face-to-face lectures (MD = 79), p < 0.001). No MCQ type assessments are held in the clinical years.

On comparing preclinical to clinical year students for SAQ the preclinical students (Md = 133) fared better with face-to-face lectures when compared to the clinical year students (Md = 93) (Mann Whitney U Test, U = 3945.0, N = 222, z = -4.529, p > 0.001). There was no difference for recorded lectures. The clinical years scored better at SAQ type assessments for core knowledge following recorded lectures (Md = 95) as compared to the preclinical year students (Md = 72) for core knowledge subjects (Mann Whitney U Test, U = 4448.5, N = 171, z = 2.993, p = 0.003).

### Discipline

Overall, grades in Prosthodontics were statistically better when assessed by MCQ following recorded lectures (Md = 75) as compared to face-to-face lectures (Md = 61, p = 0.03) and students scored statistically better results in SAQ for CK in Prosthodontics after receiving recorded lectures (Related-Samples Wilcoxon Signed Rank Test, (MD = 72), z = -2.692, n = 77, p = 0.007). Results were statistically better following face-to-face lectures in Orthodontics (Related-Samples Wilcoxon Signed Rank Test, Md = 72, z = 3.291, n = 44, p = < 0.001) and Special Care Dentistry (Related-Samples Wilcoxon Signed Rank Test, Md = 85, z = 2.158, n = 15, p = 0.031) as compared to recorded lectures (Md = 63, Md = 76, respectively). Grades achieved in assessment of applied knowledge in Operative Dentistry were statistically higher following recorded lectures (Related-Samples Wilcoxon Signed Rank Test, Md = 76, z = 2.197, n = 7, p = 0.028) as compared to face-to-face lectures (Md = 75).

### Students’ responses to open-ended questions (Q2 at T2–2020/2021)

Thoughts about Online Learning: 92% out of 98 respondents replied, of which (a) 25.6% favoured in-person lecturing since they viewed it as more engaging and allowed for student socialising. Out of these, 83% were preclinical year students; (b) 10% of respondents wanted a balanced approach between online and in-person lecturing, and; (c) 64% of respondents favoured an online approach, with (i) 21% specifically favoured pre-recorded lectures as this modality allowed for revision and viewing of the lectures at a convenient time, (ii) 2% because it kept them focused during lecturing and (iii) 41% as it allowed for better time management.

Suggested Improvements (Q2 at T2–2020/2021): 56% answered the question, of which (a) 66% reported an overall positive experience with online lecturing. Students suggested that tutors provide teaching resources in advance of the lectures so that they can prepare in advance; (b)15% reiterated the need for in-person lecturing because they view this approach as an opportunity to socialise and avoid mental health issues, and (c) 18% urged the faculty to schedule lectures better to facilitate their educational experience.

**Comments I** (Q2 at T2–2020/2021):

S17 “*Social interactions before lectures and after the lecture ends helps me personally as I can catch up with my friends and de-stress a bit. With online lectures, this idea is lost as no one wants to be there early or stay after.”.*

S62 “*Since we have clinic, it is much more convenient to have recorded lectures and follow them when we are able to dedicate the time to listen carefully, take notes and study. One would be in a more ideal state of mind, then after a tiring day of clinic. Having the lecture notes, several lectures at a time, and the ability to pause and re-listen; one can understand and build concepts in mind more effectively”.*

S86 “*Even though I prefer face-to-face lectures as I tend to grasp certain concepts better, I believe that the faculty should really look into the idea of ‘recorded lectures. I tend to understand best when watching recorded lectures since this gives us the freedom to;*

1. *Pause the video and research (the web, books etc.) since sometimes lecturers might unknowingly speed through certain concepts*.
2. *Rewatch the lecture in the future (especially prior to exams when we wish to refresh our memory)*.
3. *Watch the lecture at the most suitable time (for instance, I’m a morning person who would much prefer studying in the mornings and listening to lectures in the evenings when I tend to be more tired). On the other hand, in person lectures are important since human contact is crucial. For this reason, I believe that we should have a mix of face-to-face as well as recorded lectures”.*

S73 “*Recorded lectures could benefit students with learning disabilities, as it would allow them to view the lecture at their own pace, which is helpful if they struggle to understand the lecturer or focus during the lecture”.*

### Students’ Responses to open-ended questions (Q2 at T3–2021/2022) II

At the end of the third academic year (2021/2022), 44% of the respondents expressed no preference for changes to lecturing modes, 22% expressed a preference for a hybrid arrangement of specifically a combination of recorded and in person F2F lectures, 17% requested online lectures with a further 17% specifically requesting recorded lectures. There was one specific request for return to classroom-based lectures.

**Comments** (Q2 at T3–2021/2022) **II**:

*S61: The introduction of online lecturing is a great idea, especially with recorded lectures…. Recorded has also helped me with revision- listening at a preferred speed, stopping it at any stage, and reading and making sense of the lecture.*

*S73: Online lecturing is superb. I like best the recorded lectures and tutorials. The recorded lectures can be followed when one is best focused…* T*o be honest I was not too keen last year about it but now I really appreciated their use.*

*S77: Although I believe face to face lectures are a better and more engaging learning method than online lectures, given our hectic timetable, a mix between online lectures and face to face lectures would probably be the best option. Online lectures allow more freedom and time for studying, especially after clinical/pre-clinical sessions.*

## Discussion

Similar to other studies [14] over 74% of the students surveyed by this study requested online teaching to be retained, with a majority reporting a positive experience. Over time students reported a greater liking for online lectures, providing more efficient learning, motivation and an opportunity for self-directed learning. Furthermore, the results of this study also highlight the differences between the various teaching modalities in terms of preferences by gender, course of study and year of progression and also by adaptability to teaching content and discipline being taught.

REC lectures were statistically preferred by female students, students in their clinical years of study and students in the dental surgery course. REC lectures were the most adept at teaching core knowledge to be assessed by short answer type questions. In-person F2F lectures were initially more favoured by pre-clinical students in general and male students, this difference was then lost over time. F2F lectures were better for assessing applied knowledge. These findings reflect the reality that current student cohorts are made up of a mix of multicultural, Generation Z (born between 1995and 2010) ‘digital natives’[15] who no longer appreciate the more traditional didactic teaching and learning methods [16]. The study also serves to direct faculty when planning teaching.

This study is in accordance with previous studies that IT-based self-instructional teaching can be as effective as other methods of instruction [5]. Despite the advantages of online teaching modalities, and 82% of respondents reporting a very good/good experience with online teaching, similar to other studies [14], 22% of students surveyed by this study still favour a hybrid modality that also includes in person, face-to-face learning.

In accordance with several other studies [7, 8, 17], in this study, learning preferences were seen to be significantly influenced by gender. Females favoured multi-modal learning, including online availability of presentations and written resources, and online lectures, while males preferred the classroom experience. This is in accordance with previous literature, which showed that female students preferred a multi-modal method of learning in contrast to males, who prefer a single modal learning experience [9, 18] and that amongst those students with a single learning preference, the aural (discussions and lectures) are the most favoured [18].

In accordance with a previous study [10], a difference in student learning preferences was also identified according to the program of studies being followed. Students in the bachelor degree programs indicated a preference for presentations and written resources available on VLE to be followed by discussion sessions. The dental surgery students showed a significant preference for the availability of pre-recorded lectures to be viewed at will stating that time is better utilised for patient clinical practice. Although there is overlap in the content of the various programs of study, the dental surgery program carries a heavier component of clinical training in more varied practical skills while the bachelor programs provide more group work and group lab work. This might explain the different outlooks of the student cohorts.

Unlike previous studies [19] but in concordance with another [9], this study identified a difference in learning preferences between preclinical and clinical year students. The preclinical students significantly preferred face-to-face lecturing while the clinical year students favoured multi-modal online learning that included pre-recorded lectures, availability of various online resources, followed by tutorial sessions. Clinical year students stated that such structures were more appropriate in that they allowed for better revision of material, better preparation before discussion sessions and more motivation for self-directed learning. They strongly indicated that such blended learning was to be retained. Such differences in student outlooks could be explained by the fact that preclinical students are at a stage of fact gathering and memorising whilst clinical year students become more critical thinkers via their patient treatment experiences and have matured in their study methods. Additionally, unlike preclinical year students, clinical year students spend several additional hours on the clinical floor interacting with tutors in a face-to-face mode that allows for exchange of ideas, clarification of queries and application of knowledge.

Teachers’ enthusiasm and expressiveness have been listed as one of the seven effective teaching qualities of good educators [20]. Sub-themes of this quality include eye contact, body posture, facial expressions and language tone and humour [10]. With the increased use of online teaching modalities, there might be concern that such interaction between teacher and student is compromised. However, similar to previous studies reporting that traditional didactic teaching is no longer the preferred or most effective method [21–23], clinical year students in this study reported better communication with teachers and students over online sessions and similar learning satisfaction as with classroom learning. Additionally, tutor variables both in terms of personal interaction with students and type, quality and clarity of production of online material may explain the differences in results observed across years and disciplines.

Despite the distinct preference for online pre-recorded learning, a blended distribution of online and face-to-face/ in person teaching is to be favoured. This is to ensure contact and support for select students who expressed the need to meet classmates, familiarise themselves with tutors, and move away from the monotony of their screens. These diverse results underscore the need for Faculty to adapt their approach, in order to support and address the needs of all students [24].

The teaching of dental disciplines involves both the theoretical (core knowledge presenting principles and current facts) and the practical domains (applied knowledge presented in case-based learning formats) of the subject. The study reports that the retention of CK was significantly better following recorded lectures while that of AK was significantly better following F2F lectures. The latter facilitates tutor-student interaction and discussion that is expected to accompany topics surrounding applied knowledge; this potentially explains the results observed and underscores the need for a blended approach when tutoring students.

Limitations of the current study include that tutors vary in their interactions, modes of delivery, experience and type of preparation of online material, this could have been a source of variance not accounted for by this study.

Further studies may include evaluation of staff, faculty and administrative staff perceptions on the transition to more online learning modalities and further longitudinal analysis of student grades according to the development of further teaching modalities.

## Conclusion

This study finds that the Adult Learning theory is applicable to students of dentistry. They have shown a willingness to influence their learning and changes in curriculum, a predilection for self-directed learning and the need for freedom in engaging with resources and content. Online modalities of teaching resulted to be the preferred mode for teaching core knowledge and for dentistry clinical year students. In person F2F modes are preferred by pre-clinical year students especially those in Bachelor degree programs and in the transfer of applied knowledge. Several students also voiced the need for a blend of both in person F2F and online teaching. Such findings are important to guide faculty in aligning teaching methods of different disciplines and teaching content according to evidence based guidance and to their students’ learning preferences in a student-centred approach.

## Data Availability

The data that support the findings of this study are available from the corresponding author upon request.

## Abbreviations

AK: Applied Knowledge
CK: Core Knowledge
F2F: Face to face
MCQ: Multiple Choice Question
Q1: Questionnaire One
Q2: Questionnaire Two
REC: Recorded Lecture
SAQ: Short Answer Question
VLE: Virtual Learning Environment
